# The Role of Social Problem-Solving and Prosocial Behavior in First Aid Willingness among Adolescents in Hungary

**DOI:** 10.3390/children11060714

**Published:** 2024-06-10

**Authors:** Zsolt Katona, Klára Tarkó, Zita Petrovszki, Ferenc Győri, Beáta Vári, Balázs Polcsik, Tamás Berki

**Affiliations:** 1 Institute of Physical Education and Sport Sciences, Juhász Gyula Faculty of Education, University of Szeged, 6725 Szeged, Hungary; katona.zsolt@szte.hu (Z.K.); hajdune.petrovszki.zita@szte.hu (Z.P.); vari.beata@szte.hu (B.V.); polcsik.balazs@szte.hu (B.P.); 2 MTA-SZTE Health Promotion Research Group, 6725 Szeged, Hungary; tarko.klara@szte.hu; 3 Institute of Applied Health Sciences and Environmental Education, Juhász Gyula Faculty of Education, University of Szeged, 6725 Szeged, Hungary; 4 Institute of Physiotherapy and Sports Science, Faculty of Health Science, University of Pécs, 7621 Pécs, Hungary; ferenc.gyori@etk.pte.hu; 5 Sport Science Research Group, Gál Ferenc University, 6720 Szeged, Hungary; 6 Department of Physical Education Theory and Methodology, Hungarian University of Sports Science, 1123 Budapest, Hungary

**Keywords:** bystander, social competence, youth, help, peers

## Abstract

First aid is a key factor in preventing further deterioration in an accident, saving lives, or improving treatment in emergencies. However, the reasons behind the willingness to provide first aid are still unclear. Therefore, this study aimed to investigate the role of social problem-solving and prosocial behavior in the dimension of first aid willingness. Self-administered questionnaires were used to evaluate the dimensions of first aid willingness (first aid willingness for peers, first aid willingness for strangers, knowledge, and negative emotions), social problem solving (positive problem orientation, negative problem orientation, rational problem solving, avoidance style, and impulsivity/carelessness) and prosocial behavior. A total of 497 school-aged students between the ages of 12 and 15 years (201 boys and 296 girls) participated in this study. Our results showed that positive problem orientation (*p* < 0.05) and rational problem solving (*p* < 0.001) are significant factors in determining first aid willingness for both peers and strangers. On the other hand, avoidance style orientation (*p* < 0.05) has a negative influence on the willingness to provide first aid to peers and strangers. Negative problem orientation (*p* < 0.001) only predicted negative emotions related to emergencies. Furthermore, prosocial behavior (*p* < 0.001) was more strongly associated with first aid willingness than social problem solving. Our study suggested that improving social competence could be a key factor in increasing first aid provision in real time, which could save lives in emergencies.

## 1. Introduction

Timely and skilled first aid can prevent further deterioration of the victim’s condition, potentially save lives, and improve overall treatment and rehabilitation [1]. Studies emphasize the importance of training individuals, like adolescents or adults, in first aid to improve their knowledge and skills in assisting accident victims [2,3,4]. Furthermore, a lack of immediate first aid can make situations critical and lead to increased complications and secondary injuries [5,6]. In order to mitigate the harmful effects of accidents and improve the outcome of injuries, it is also essential to popularize first aid training and ensure the preparedness of different groups [7]. Previous studies have suggested that one of the focus groups on first aid willingness should target adolescents, as this could help to increase the quality of first aid and shorten the time gap for providing proper help [8,9,10]. Therefore, it is important to understand adolescents’ attitudes toward first aid and what behaviors motivate them to help others. We believe that the willingness to provide first aid not only depends on knowledge but also on social factors such as prosocial behavior and social problem solving; hence, the current study investigates the influence of social competence factors on first aid willingness.

**Figure 1:**
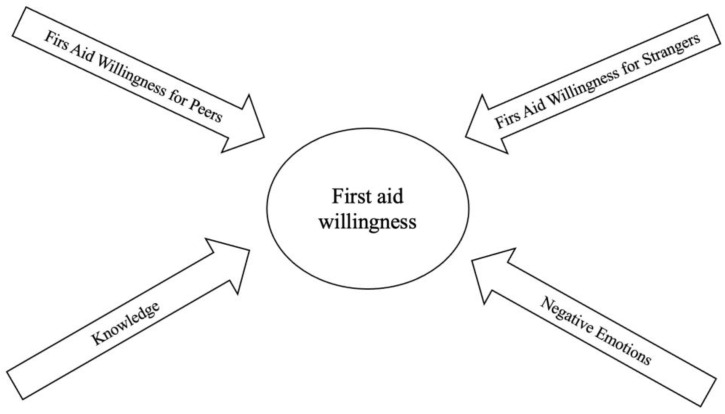
The dimension of first aid willingness.

In our previous study, we examined the factors that can influence individuals’ willingness to provide first aid [9]. The study revealed that first aid willingness has four dimensions: first aid willingness for peers, first aid willingness for strangers, knowledge, and negative emotions (as shown in Figure 1).

First aid willingness for peers refers to the behavior in which individuals are willing to help their friends, families, classmates, and others. On the other hand, first aid willingness for strangers refers to the attitude that an individual will have toward helping a stranger. Knowledge refers to previous experience and training regarding first aid, which is also highly correlated with first aid willingness [9]. Negative emotions, such as disgust and fear of unsuccessful intervention, can also hinder giving first aid. Although only a few studies have investigated first aid willingness, it seems that it is mainly correlated with knowledge, self-efficacy, and previous experience [9,11,12,13]. Furthermore, studies showed that adolescents are more willing to provide first aid to their peers, as they have decreased stigmatizing attitudes [9,14]. Moreover, girls and older individuals have an increased willingness to help as well [9,14]. Previous studies have found that girls have a more empathic attitude than males in helping behavior, and they react more strongly and are more supportive in these situations [15,16,17]. The role of age in helping is mainly associated with knowledge and training in first aid, since older individuals are more likely to have had previous first aid experience. Other studies also suggest that as individuals mature, they may develop a greater sense of social responsibility [18,19,20].

Social behavior might be an important factor that affects first aid willingness. Previous studies found that people hesitate to act in groups in an emergency due to the diffusion of responsibility [21,22]. Thus, investigating social behavior is the key to understanding more about first aid willingness. Social competence is a widely used framework for understanding the underlying behavioral factors of social behavior. Multiple models have been identified for social competencies over the years [23,24,25]. These models suggest that social competence is a multidimensional model with three dimensions (cognitive, competence, and affective) [23,26].These dimensions can be divided into inherited and learned components such as ability, skill, motive, habit, knowledge, pattern, tendency, etc. [23].

Social problem solving is the cognitive part of social competence and is defined as a “self-directed cognitive-behavioral process by which a person attempts to identify or discover adaptive ways of coping with problematic situations encountered in everyday living” [27] (p. 242). According to D’Zurilla’s model, there are two main aspects of social problem solving. First, problem orientation is the motivational part of the problem-solving process. It comprises a set of relatively stable cognitive–emotional schemas that describe how a person generally thinks and feels about problems in life, as well as their problem-solving ability (e.g., challenge and threat appraisals, problem-solving self-efficacy beliefs, outcome expectancies). Second, problem-solving proper, on the other hand, refers to the search for a solution through the rational application of specific problem-solving skills that are designed to maximize the probability of finding the “best” or most adaptive solution to a given problem. Previous studies by D’Zurilla [28] have shown that the two main concepts can be categorized into five different factors. Problem orientation has been divided into positive and negative problem orientations (NPO), where positive problem orientation (PPO) refers to those cognitive constructs that contribute to believing in individual problem-solving capacities, devoting time and effort to helping others, and expecting positive outcomes for the problem [28]. NPO refers to dysfunctional cognitive–emotional constructs that indicate doubts about individual problem-solving abilities and negative problem-solving outcomes. Three factors were identified regarding problem-solving proper: rational problem solving (RPS), impulsivity/carelessness (ICS), and avoidance style (AS). RPS refers to a constructive cognitive behavior pattern that involves the conscious and systematic application of certain problem-solving skills (e.g., defining and searching for alternative solutions). The ICS assesses deficient cognitive behavior patterns characterized by impulsive, careless, hasty, and incomplete efforts to apply problem-solving strategies and techniques. AS refers to a faulty pattern of behavior that involves a tendency to postpone problem solving as long as possible, waiting for problems to solve themselves, and shifting responsibility for problem solving to others [28].

Several studies have investigated social problem-solving associations. For example, previous studies have shown that social problem solving is correlated with stress, a positive attitude toward seeking mental health, memory, decreased depression, and well-being [29,30,31,32,33,34]. Additionally, personality was also found to be a significant predictor. Personal traits such as extraversion, openness, conscientiousness, agreeableness, and self-esteem positively correlate with social problem solving, while neuroticism shows a negative correlation [35]. To the best of our knowledge, no previous study has examined the relationship between social problem solving and willingness to provide first aid, but helping others may be associated with problem-solving skills and improved interpersonal relationships and communication [36].

Prosocial behavior is an important aspect of social competence. It involves voluntary actions aimed at benefiting others [37,38]. Research suggests that prosocial behavior is related to peer acceptance and intellectual competencies and can enhance empathy, moral reasoning, and affective functioning [39,40,41,42]. It is also associated with social problem-solving abilities. Previous studies found that individuals with strong social problem-solving abilities are more likely to engage in prosocial behaviors, as they can navigate social situations, understand others’ perspectives, and find constructive solutions to conflicts [43]. Prosocial behavior plays a crucial role in helping others as well. Previous studies have shown that bystander effects are also important. “The bystander effect refers to the phenomenon that an individual’s likelihood of helping decreases when passive bystanders are present in a critical situation” [44] (p. 517). For example, studies indicate a significant positive relationship between the bystander effect and prosocial behavior among psychology students, emphasizing the importance of prosocial tendencies in influencing bystander actions [45]. Moreover, priming the concept of prosociality has been found to counteract the bystander effect, increasing responsiveness to requests for help even in the presence of many bystanders [46]. These findings suggest that prosocial behavior is key to shaping bystander responses across different scenarios and can contribute to the well-being of societies.

Based on research on the role of problem solving in interpersonal relationships, personal traits, and prosocial behavior in bystander responses [35,36,45,46], we expect that first aid willingness may be associated with these factors. Our study aimed to investigate the role of social problem-solving and prosocial behavior in the dimension of first aid willingness. Furthermore, our study objective includes understanding the influence of gender differences on social problem-solving, prosocial behavior, and the dimension of first aid willingness. To the best of our knowledge, no previous studies have explored this phenomenon. Additionally, we aimed to validate the first aid willingness questionnaire developed by Katona and his colleagues [9]. Due to the positive correlations with positive attitudes and decreased stress, we hypothesize that a positive problem-solving orientation will predict all dimensions of first aid willingness, except for negative emotions. On the other hand, a negative problem-solving orientation positively predicts negative emotions [29,30]. We also hypothesize that problem-solving skills, such as rational problem solving, will positively predict the dimensions of first aid willingness, while impulsivity/carelessness and avoidance will negatively affect the dimensions of first aid willingness. We believe that prosocial behavior has a positive influence on the dimension of first aid willingness since previous studies emphasize its positive relationship with bystander effects [45].

## 2. Materials and Methods

### 2.1. Participants and Procedure

Our research data were collected between the fall of 2023 and the spring of 2024. We used an online self-administered questionnaire in Hungarian to evaluate social competence factors and willingness to provide first aid. The questionnaire was sent to schools, and we visited some of them in person to help with the process. In the beginning of our research, we contacted nine local schools, and four of them answered and participated in our study. The procedure was the same in all schools. At first, the school principals approved our questionnaire and the goal of the study, then the parents were informed, who provided parental consent to allow their children to participate in this study. It is important to note that during the data collection, we did not provide any first aid training to the respondents. The online questionnaires were filled out in school classes via tablets or their phones. Before data collection, all the students were informed about the study, and they were assured that their participation was voluntary and no personal data such as names were collected. Furthermore, all participants provided online informed consent to participate in the study. The study was approved ethically by the Institutional Review Board of the University of Szeged (Ethical Approval Number: 12/2022).

### 2.2. Measures

#### 2.2.1. Sociodemographic and First Aid Background

During the study, students were asked to provide sociodemographic data such as their age, gender, place of residence, and family financial status. Moreover, they were also asked to provide additional information regarding their previous experience with first aid, such as first aid education (e.g., “Have you learned any first aid skills?”). We asked the participants if they had ever been given immediate first aid after an accident (“Have you ever received immediate first aid in your life?”) and if they had ever provided first aid to others (e.g., friends or family members).

#### 2.2.2. First Aid Willingness Questionnaire

First aid willingness was assessed using the so-called First Aid Willingness Questionnaire [9]. The scale consisted of 20 items, and the answer categories were on a 5-point Likert-type scale (1 = strongly disagree to 5 = strongly agree). The questionnaire included four subscales. First aid willingness for peers comprised five items and scenarios that involved friends and family (e.g., my brother falls while skating, I help him immediately). First aid willingness for strangers is meant when strangers are concerned (e.g., an old man faints in the street; I notice him and call for help.). Negative emotions refer to negative feelings regarding giving first aid (e.g., I would be afraid to help someone lying unconscious). The subscale of knowledge included items that refer to all the knowledge that individuals have about first aid (e.g., an adult woman lies unconscious in the corner; I know how to perform CPR). The questionnaire was initially developed in an earlier study [9]. However, in this study, the scale was developed further. The original version included 16 items, and four additional items were added to enhance construct validity and reliability.

#### 2.2.3. Social Problem-Solving Inventory

The Social Problem-Solving Inventory-Revised Short-Form was utilized to investigate the social problem-solving skills of the sample. The scale was originally developed by D’Zurilla and his colleagues [27] and was adapted to Hungarian culture by Kasik [47]. The inventory consisted of 25 items and was designed to measure an individual’s cognitive, affective, and behavioral responses to real-life situations. It included five subscales: positive problem orientation (PPO), negative problem orientation (NPO), rational problem solving (RPS), impulsivity/carelessness style (ICS), and avoidance style (AS). Each subscore contains five items that are rated on a five-point Likert-type scale ranging from 0 (not at all true) to 4 (extremely true). Higher subscores for PPO and RPS and lower subscores for NPO, ICS, and AS indicate good social problem-solving abilities.

#### 2.2.4. Prosocial Behavior

Prosocial behaviors were measured by Kóródi and her colleague’s prosocial behavioral scale [48]. The scale consisted of 10 items about individuals’ prosocial behavior (e.g., “I notice when someone is in trouble”). The answer options were on a five-point Likert-type scale (1 = never to 5 = always). The scale was unidimensional and had no subscales.

### 2.3. Statistical Analysis

All the statistical analyses were performed via Jamovi 2.3 for Mac. First, primary analysis was conducted using confirmatory factor analysis on the first aid willingness questionnaire, social problem-solving inventory, and prosocial behavioral scale to determine the construct validity and internal reliability of the scale. The following fit indices were used: chi-square (x^2^), relative chi-square divided by the degree of freedom (CMIN/d.f), root mean square error of approximation (RMSEA), a nonnormed fit index called the Tucker–Lewis index (TLI), comparative fit index (CFI), and standardized root mean square residual (SRMR). The acceptable range of the fit indices was taken from the previous literature [49,50,51]. Three widely used coefficients were used to test the internal reliability of our questionnaire: average variance extracted (AVE), composite reliability (CR), and Cronbach alpha. The acceptable range of the reliabilities was the following: AVE = 0.50; CR = 0.70; Cronbach alpha = 0.70 [52].

Descriptive statistics were used to see the sample’s characteristics. Multivariate Analysis of Covariance (MANCOVA) and Analysis of Covariance (ANCOVA) with partial eta squared (ηp^2^) were used to determine the gender differences. The criteria for ηp^2^ were the following: 0.01 indicates a small effect; 0.06 indicates a medium effect; and 0.14 indicates a large effect [53]. Furthermore, Pearson correlations were used to measure the strengths and directions between the variables. The mean values of the psychometric questionnaires used ranged from 1 to 5 in this study.

Hierarchal multiple regression was used as the main analysis method to determine the relationships among the factors of first aid willingness, social problem solving, and prosocial behavior. Each of the factors from the first aid willingness questionnaire (first aid willingness for peers, first aid willingness for strangers, knowledge, and negative emotions) was set as an outcome variable separately in the models, and the following model was tested. In the first step (Model 1), control variables, such as gender and age, were added; then, in the second step (Model 2), PPO, RPS, NPO, ICS, and AS were added. Finally, prosocial behavior was added in the third step (Model 3). We decided to add prosocial behavior in the last step of the model because previous studies suggest that prosocial behavior can be considered an outcome of effective social problem-solving skills [43].

## 3. Results

### 3.1. The Participants

Our sample consisted of 497 school-aged students (boys = 201; girls = 296) between the ages of 12 and 15. The average age of the participants was 13.45 years (SD = 1.20). The average age of the boys was 13.48 years (SD = 1.12), and that of the girls was 13.43 years (SD = 1.26). Table 1 presents the sample characteristics, which include sociodemographic information and previous first aid experience.

### 3.2. Primary Analysis

Confirmatory factor analysis was used as a primary analysis to verify the structural models of the first aid willingness questionnaire. In our previous study, four factors were identified using exploratory confirmatory analysis regarding the first aid willingness questionnaire (first aid willingness for peers, first aid willingness for strangers, knowledge, and negative emotions) [9]. Hence, the model was built on these results. The scale showed an excellent model fit (x^2^(152) = 354.45, *p* = 0.01; CMIN/d.f. = 2.33; CFI = 0.95; TLI = 0.93; SRMR = 0.06; RMSEA = 0.05). All subscales included five items, and the factor loads varied between 0.62 and 0.83.

The confirmatory factor analysis was also conducted on the social problem-solving inventory and prosocial behavioral scale. The five factors of social problem-solving inventory showed a good fit in this study (x^2^(213) = 564.99, *p* = 0.01; CMIN/d.f. = 2.65; CFI = 0.91; TLI = 0.90; SRMR = 0.06; RMSEA = 0.06), and the factor loads varied between 0.53 and 0.83. The prosocial behavioral scale was a unidimensional scale with an excellent model fit (x^2^(31) = 71.73, *p* = 0.01; CMIN/d.f. = 2.32; CFI = 0.97; TLI = 0.95; SRMR = 0.03; RMSEA = 0.05), and the factor loads were between 0.43 and 0.82. The internal consistencies were measured using three methods (AVE, CR, and Cronbach alpha), which can be seen in Table 2 for all scales.

### 3.3. Descriptive and Gender Differences

Table 3 presents the means, standard deviations, and gender differences for the observed variables. The highest score was for first aid willingness toward strangers (M = 3.76), whereas the lowest score was for AS (M = 2.47). Regarding social problem solving, PPO (M = 3.67) and RPS (M = 3.59) had the highest scores. Prosocial behavior had a mean score of 3.09. The multivariate analysis of covariance (MANCOVA) was conducted to investigate the gender differences controlling for age on the factors of first aid willingness, prosocial behavior, and social problem solving. The multivariate tests showed significant differences between genders in the combined dependent variables while controlling for age (Wilks’ Λ = 0.92, F(9) = 4.85, *p* = 0.00, ηp^2^ = 0.14). Since gender and age had a significant role in the variables of first aid willingness, prosocial behavior, and social problem solving, we further analyzed them in this study and added them as a control variable in our regression models. A univariate test adjusted for age (ANCOVA) showed that negative emotions (*p* < 0.05), NPO (*p* < 0.001), and prosocial behavior (*p* < 0.001) were significantly greater for girls, while PPO (*p* < 0.001) and ICS (*p* < 0.05) were significantly greater for boys. In all cases, ηp^2^ showed low effects [53].

### 3.4. Bivariate Correlations

The pattern of the bivariate correlations of age, the dimension of first aid willingness, factors of social problem solving, and prosocial behavior were expected (Table 4). Age had small correlations with the study variable. PPO, RPS, and prosocial behavior were positively correlated with first aid willingness for peers, first aid willingness for strangers, knowledge, and negative emotions. NPO and AS had negative associations with first aid willingness for peers, first aid willingness for strangers, and knowledge, and positive associations with negative emotions.

### 3.5. Hierarchical Multiple Regression Analysis

The results of the hierarchical multiple regression analysis can be found in Table 5. Each subscale of the first aid willingness questionnaire was analyzed separately. In Model 1, age and gender were added to the model as control variables in each case. Age was found to be a significant predictor in most cases (except for first aid willingness for peers), and gender was only a significant predictor of negative emotions. The subscales of the Social Problem-Solving Inventory were added in the second step. PPO (β = 0.15), RPS (β = 0.19), and AS (β = −0.15) were significant predictors of first aid willingness for peers. Similarly, PPO (β = 0.13), RPS (β = 0.22), and AS (β = −0.15) significantly predicted first aid willingness for strangers. NPO significantly predicted negative emotions (β = 0.43). RPS (β = 0.26) significantly predicted knowledge. Finally, prosocial behavior was added to the model (step 3). First aid willingness for peers was significantly predicted by prosocial behavior (β = 0.55). First aid willingness for strangers is significantly associated with age (β = 0.11), RPS (β = 0.10), and prosocial behavior (β = 0.46). Age (β = −0.10) and prosocial behavior (β = −0.15) predicted negative emotions. Knowledge was influenced by age (β = 0.16), RPS (β = 0.17), and prosocial behavior (β = 0.40). The variance is explained by 32% of the highest variance and 21% of the lowest variance in the model.

## 4. Discussion

The goal of this research was to investigate the role of social problem-solving and prosocial behavior in the dimension of first aid willingness. In addition, we aimed to examine the reliability and validity of a questionnaire that measures first aid willingness. Our findings indicate that the first aid willingness questionnaire is a reliable tool for evaluating the dimension of first aid willingness. Furthermore, our study demonstrated that prosocial behavior is more crucial than social problem solving in determining the dimension of first aid willingness.

As a primary analysis, we investigated the structure of the first aid willingness questionnaire. In our previous study, we identified four factors related to first aid willingness that are important for helping other adolescent students [9]. The original scale was expanded and revised, and the final version had an excellent model fit, making it suitable for adolescents aged 12–15 years. Three reliability values were used to determine the scale reliability. When the AVE was lower than expected, the CR value was considered more appropriate for reliable conclusions. According to Fornell and Larcker [54], if the AVE is less than 0.5 but the composite reliability is higher than 0.6, the construct’s reliability can be adequate [54,55]. Therefore, we relied on acceptable Cronbach’s alpha and CR values, and our reliability values were within the adequate range. We initially aimed to gain a better understanding of the first aid willingness questionnaire. However, during our analysis, we also examined the structure and reliability of the social problem-solving inventory and the prosocial behavioral scale. Our findings indicated that these scales functioned effectively within this sample, similar to our observations with the first aid willingness questionnaire.

In our main analysis, hierarchal multiple regression analysis was used to investigate the effect of social problem solving and prosocial behavior on first aid willingness. Age and gender were added as the control variables in our model, but they had minor effects on our results. We must acknowledge that our primary analysis also showed minor effects regarding age and gender. As we saw, gender did not significantly predict any dimension of first aid willingness when the main variables were included in the model, suggesting that the dimension of first aid willingness is not dependent on gender in this sample. The primary analysis only showed gender differences while controlling for age in negative emotions, but it also showed minor effects and small differences. We must acknowledge that gender could be an important predictor, since girls have a more empathetic attitude and are more supportive in helping situations [15,16,17]. Investigating only age was found to be positively correlated with first aid willingness for strangers and knowledge and negatively with negative emotions. Older students had more knowledge, were more willing to help others, and experienced fewer negative emotions. Previous studies have highlighted the positive influence of first aid training on willingness to provide first aid [56,57]. Since first aid training is more likely for older age groups, this could explain their greater knowledge and willingness to help, which in turn reduces negative thoughts [8,9,10]. Overall, it seems that both age and gender have a minor effect on our results, but aging seems to help first aid willingness, maybe due to personal development.

Positive problem orientation was a significant and positive predictor of willingness to provide first aid to both peers and strangers. Positive problem orientation is the motivational aspect of social problem solving. Individuals with a high positive problem orientation are more likely to believe that a problem can be solved, and they are motivated to do so. Our results were in line with our hypotheses, and they indicate that the first step in an emergency is the motivation to help. Furthermore, we believe that there are indirect effects on these results. As we have previously observed, social problem-solving skills are positively associated with personal traits such as openness and conscientiousness [35]. For instance, conscientiousness is linked to cognitive function and helps individuals be organized and goal-directed, which is crucial during emergency situations [58]. We believe that other personal traits also indirectly affect first aid willingness, but further analysis is required to understand this phenomenon. Additionally, positive problem orientation was found to be correlated with knowledge of first aid, suggesting that increasing knowledge could also increase motivation to provide first aid. On the other hand, negative problem orientation only predicted negative emotions. This result was expected and in line with our hypotheses since negative problem orientations are characterized by cognitive dysfunction, in which individuals do not believe they can help others. As a result, their negative emotions increase in such situations.

Rational thinking seems to increase the willingness to provide first aid to both peers and strangers. Furthermore, it was also associated with knowledge. Rational thinking is a cognitive function that is interconnected with knowledge [59]. It involves a conscious and systematic approach that can aid in providing first aid since first aid solutions are based on different protocols [60]. Additionally, in emergencies, rational problem solving can be advantageous since it helps with decision-making and stronger decisions [61]. Moreover, a study by Harmon [62] revealed that rational problem solving works as a coping mechanism for emergencies, which could explain our results.

The avoidance style has been found to have a negative influence on the willingness to provide first aid to both peers and strangers. This is not surprising, as the avoidance style involves avoiding problem solving, which is a dysfunctional behavior. People with a high avoidance style tend to postpone taking responsibility for their problems and finding solutions, which negatively affects their willingness to provide first aid to others. This phenomenon has been supported by research conducted by Eskin and his colleagues [60].

We included prosocial behavior as a predictor in our final model, and it was found to be strongly associated with first aid willingness. We must acknowledge that when the variable of prosocial behavior was added to the model, all the factors related to social problem solving decreased, indicating a significant influence of prosocial behavior on first aid willingness. This result was expected since prosocial behavior was found to be crucial in helping others and influencing bystanders’ actions [45]. However, it was not expected that it would lower the effects on social problem solving. We believe these results were due to the affective responses associated with prosocial behavior. For example, previous studies have shown the role of empathy in prosocial behavior, which can contribute to first aid willingness and helping others [39,63]. Furthermore, other researchers have found that prosocial behavior increases affective decisions [64]. These findings could explain why only prosocial behavior was significant in the final model of first aid willingness for peers. Only negative emotions negatively predict prosocial behavior, meaning that prosocial behavior might be an important protector of negative emotions.

Interestingly, impulsivity and carelessness did not predict first aid willingness in any of our participants. As previously mentioned, impulsivity and carelessness refer to a pattern of deficient cognitive behavior characterized by impulsive, hasty, and incomplete efforts to solve problems [28]. Moreover, it is associated with attitudes such as a lack of perseverance and lack of premeditation, which are not advantageous during an accident [65]. Although negative effects were hypothesized, the results were not surprising.

Our study has several limitations that need to be addressed. First, we must acknowledge that we used self-administered questionnaires with convenient sampling that could cause bias. Second, we need to consider the limitations with regard to the dimension of first aid willingness. Only a few studies have investigated this topic, and therefore, the generalizability of the findings is limited; hence, conclusions can only be drawn for this sample. We must acknowledge that the first aid willingness questionnaire is only reliable between ages 12 and 15, which also limits its generalizability. Our goal is to overcome these limitations by adding more variables to increase the generalizability of the dimension of first aid willingness and by adding more age groups to increase the sample size.

## 5. Conclusions

To summarize our research, we found that having a positive problem orientation is crucial for showing willingness to provide first aid to peers and strangers. Conversely, an avoidance style has a negative influence on first aid willingness in such situations. Moreover, we found that prosocial behavior has a stronger association with first aid willingness than does social problem solving. Finally, we successfully developed a reliable questionnaire that could help researchers investigate the dimension of first aid willingness. Hence, we encourage researchers worldwide to help expand the generalizability of our questionnaire and understand more about the willingness to administer first aid, which could save lives in emergencies.

## Figures and Tables

**Table 1:** Characteristics of the sample.

	n	%
Gender		
Boy	201	40%
Girl	296	60%
Place of residence		
Village	80	16%
Small town	64	12%
City	296	60%
Capital	57	12%
Financial status		
Upper-class	20	5%
Upper-middle-class	127	34%
Middle-class	201	53%
Lower-middle-class	29	8%
Accident following first aid		
Yes	232	47%
No	265	53%
Giving first aid ever		
Yes	60	12%
No	437	88%
First aid learning		
Yes	238	48%
No	259	52%
Place of first aid learning		
School	191	80%
Outside of school (e.g., camp)	33	14%
Family	14	6%

**Table 2:** Reliabilities of the study measures.

	Cronbach Alpha	AVE	CR
*First Aid Willingness Questionnaire*			
First Aid Willingness for Peers	0.82	0.47	0.81
First Aid Willingness for Strangers	0.78	0.43	0.79
Negative Emotions	0.72	0.36	0.73
Knowledge	0.80	0.38	0.78
*Social Problem Solving Inventory*			
PPO	0.71	0.41	0.73
NPO	0.81	0.43	0.79
RPS	0.73	0.41	0.73
ICS	0.75	0.64	0.76
AS	0.83	0.50	0.83
*Prosocial Behavioral Scale*	0.82	0.43	0.82

Note: PPO = Positive Problem Orientation; NPO = Negative Problem Orientations; RPS = Rational Problem Solving; ICS = Impulsivity/Carelessness; AS = Avoidance Style.

**Table 3:** Means, standard deviations, and gender differences for the observed variables.

	Total (M; SD)	Boys (M; SD)	Girls (M; SD)	F-Test	ηp^2^
First Aid Willingness for Peers	3.69 (0.87)	3.65 (0.87)	3.71 (0.86)	0.49	0.00
First Aid Willingness for Strangers	3.76 (0.82)	3.68 (0.87)	3.81 (0.78)	3.06	0.00
Negative Emotions	2.9 (0.88)	2.78 (0.88)	2.98 (0.87)	5.95 *	0.01
Knowledge	3.36 (0.9)	3.38 (0.9)	3.35 (0.89)	0.10	0.00
PPO	3.67 (0.75)	3.8 (0.64)	3.58 (0.81)	10.58 ***	0.02
NPO	2.45 (0.93)	2.26 (0.84)	2.58 (0.97)	14.13 ***	0.02
RPS	3.59 (0.79)	3.59 (0.81)	3.6 (0.78)	0.00	0.00
ICS	2.90 (0.8)	3.00 (0.78)	2.84 (0.8)	5.28 *	0.01
AS	2.47 (0.98)	2.49 (0.94)	2.45 (1.01)	0.17	0.00
Prosocial Behavior	3.09 (0.55)	2.95 (0.55)	3.19 (0.52)	25.52 ***	0.04

Note: * *p* < 0.05; *** *p* < 0.001. PPO = Positive Problem Orientation; NPO = Negative Problem Orientations; RPS = Rational Problem Solving; ICS = Impulsivity/Carelessness; AS = Avoidance Style.

**Table 4:** Bivariate correlations for the observed variables.

	1.	2.	3.	4.	5.	6.	7.	8.	9.	10.
1. Age	—									
2. First Aid Willingness for Peers	−0.01	—								
3. First Aid Willingness for Strangers	0.11 *	0.75 ***	—							
4. Negative Emotions	−0.11 *	−0.15 ***	−0.14 **	—						
5. Knowledge	0.17 ***	0.65 ***	0.73 ***	−0.21 ***	—					
6. PPO	0.12 **	0.29 ***	0.24 ***	−0.23 ***	0.24 ***	—				
7. NPO	−0.07	−0.16 ***	−0.09	0.42 ***	−0.14 **	−0.59 ***	—			
8. RPS	0.08	0.27 ***	0.29 ***	−0.10 *	0.30 ***	0.47 ***	−0.24 ***	—		
9. ICS	−0.01	−0.03	−0.04	0.13 **	−0.03	−0.05	0.27 ***	−0.34 ***	—	
10. AS	−0.02	−0.24 ***	−0.21 ***	0.23 ***	−0.14 **	−0.53 ***	0.60 ***	−0.33 ***	0.34 ***	—
11. Prosocial Behavior	−0.02	0.55 ***	0.52 ***	−0.12 **	0.42 ***	0.30 ***	−0.08	0.34 ***	−0.05	−0.28 ***

Note: * *p* < 0.05; ** *p* < 0.01; *** *p* < 0.001. PPO = Positive Problem Orientation; NPO = Negative Problem Orientations; RPS = Rational Problem Solving; ICS = Impulsivity/Carelessness; AS = Avoidance Style.

**Table 5:** Results of the hierarchical multiple regression analysis for the predictors of first aid willingness.

		First Aid Willingness for Peers β (SE)	First Aid Willingness for Strangers β (SE)	Negative Emotions β (SE)	Knowledge β (SE)
**Model 1**					
	Gender ^a^	0.06 (0.08)	0.08 (0.07)	0.11 * (0.08)	−0.01 (0.08)
	Age	−0.00 (0.03)	0.11 * (0.03)	−0.11 * (0.03)	0.17 *** (0.03)
**Model Summary**	R^2^	0.00	0.02	0.02	0.03
	ΔR^2^	0.00	0.00	0.00	0.00
	F	0.26	4.37 *	6.30 **	7.42 ***
**Model 2**					
	Gender ^a^	0.05 (0.08)	0.08 (0.07)	0.04 (0.08)	0.01 (0.01)
	Age	−0.04 (0.03)	0.08 (0.03)	−0.09 * (0.03)	0.14 * (0.04)
	PPO	0.15 * (0.07)	0.13 * (0.05)	0.03 (0.07)	0.08 (0.15)
	NPO	0.03 (0.06)	0.11 (0.05)	0.43 *** (0.06)	−0.04 (0.01)
	RPS	0.19 *** (0.06)	0.22 *** (0.05)	0.00 (0.06)	0.26 *** (0.31)
	ICS	0.09 (0.05)	0.07 (0.05)	0.03 (0.05)	0.08 (0.07)
	AS	−0.15 * (0.06)	−0.15 * (0.05)	−0.02 (0.05)	−0.01 (−0.02)
**Model Summary**	R^2^	0.13	0.13	0.19	0.12
	ΔR^2^	0.13	0.11	0.16	0.12
	F	10.06 ***	10.44 ***	16.29 ***	9.95 ***
**Model 3**					
	Gender ^a^	−0.06 (0.07)	−0.03 (0.07)	0.08 (0.08)	−0.08 (0.08)
	Age	−0.01 (0.03)	0.11 * (0.03)	−0.10 * (0.03)	0.16 *** (0.03)
	PPO	0.04 (0.06)	0.03 (0.06)	0.17 (0.07)	−0.02 (0.07)
	NPO	−0.06 (0.05)	0.02 (0.05)	0.46 *** (0.06)	−0.10 (0.05)
	RPS	0.06 (0.05)	0.10 * (0.05)	0.04 (0.06)	0.17 *** (0.06)
	ICS	0.04 (0.05)	0.03 (0.05)	0.04 (0.05)	0.04 (0.05)
	AS	−0.04 (0.55)	−0.05 (0.04)	−0.05 (−0.05)	0.07 (0.05)
	Prosocial Behavior	0.51 *** (0.07)	0.46 *** (0.07)	−0.15 * (−0.07)	0.40 *** (0.07)
**Model Summary**	R^2^	0.32	0.30	0.21	0.25
	ΔR^2^	0.20	0.17	0.02	0.13
	F	29.21 ***	26.49 ***	15.20 ***	19.94 ***

Note: * *p* < 0.05; ** *p* < 0.01; *** *p* < 0.001. ^a^ Gender: 1 = boys; 2 = girls. PPO = Positive Problem Orientation; NPO = Negative Problem Orientations; RPS = Rational Problem Solving; ICS = Impulsivity/Carelessness; AS = Avoidance Style.

## Data Availability

The data presented in this study are available on request from the corresponding author. The data are not publicly available due to privacy reasons.
